# Predicting Lung Cancer in the United States: A Multiple Model Examination of Public Health Factors

**DOI:** 10.3390/ijerph18116127

**Published:** 2021-06-06

**Authors:** Arnold Kamis, Rui Cao, Yifan He, Yuan Tian, Chuyue Wu

**Affiliations:** International Business School, Brandeis University, 415 South Street, Waltham, MA 02454-9110, USA; ruicao@brandeis.edu (R.C.); yifanhe@brandeis.edu (Y.H.); yuantian@brandeis.edu (Y.T.); chuyuewu@brandeis.edu (C.W.)

**Keywords:** adult smoking, lung cancer, united states, regression, environmental quality index, ambient emissions, machine learning, transparency, iterative modeling

## Abstract

In this research, we take a multivariate, multi-method approach to predicting the incidence of lung cancer in the United States. We obtain public health and ambient emission data from multiple sources in 2000–2013 to model lung cancer in the period 2013–2017. We compare several models using four sources of predictor variables: adult smoking, state, environmental quality index, and ambient emissions. The environmental quality index variables pertain to macro-level domains: air, land, water, socio-demographic, and built environment. The ambient emissions consist of Cyanide compounds, Carbon Monoxide, Carbon Disulfide, Diesel Exhaust, Nitrogen Dioxide, Tropospheric Ozone, Coarse Particulate Matter, Fine Particulate Matter, and Sulfur Dioxide. We compare various models and find that the best regression model has variance explained of 62 percent whereas the best machine learning model has 64 percent variance explained with 10% less error. The most hazardous ambient emissions are Coarse Particulate Matter, Fine Particulate Matter, Sulfur Dioxide, Carbon Monoxide, and Tropospheric Ozone. These ambient emissions could be curtailed to improve air quality, thus reducing the incidence of lung cancer. We interpret and discuss the implications of the model results, including the tradeoff between transparency and accuracy. We also review limitations of and directions for the current models in order to extend and refine them.

## 1. Introduction

Worldwide, lung (and bronchus) cancer is the most common cancer. It is the second most common type in the United States, and cancer overall was the number two cause of death in 2019, slightly behind heart disease (599,601 vs. 659,041) [1]. Although the incidence of lung cancer has been decreasing steadily, it remains the leading cause of death from cancer. In 2020 in the United States, estimated new cases were 228,820 and estimated deaths were 135,720.

Cigarette smoking has been decreasing slowly but steadily because of public service announcements, creative anti-smoking campaigns, and bans of smoking in many business establishments. In 2021, however, there are estimated to be 235,760 new cases of lung cancer in the United States, with men having a slightly higher rate than women. There are racial disparities as well, with black men about 15% more likely to develop cancer than white men. Lung cancer survival is better for Hispanics. Smoking raises the risk substantially for everyone.

**Figure 1:**
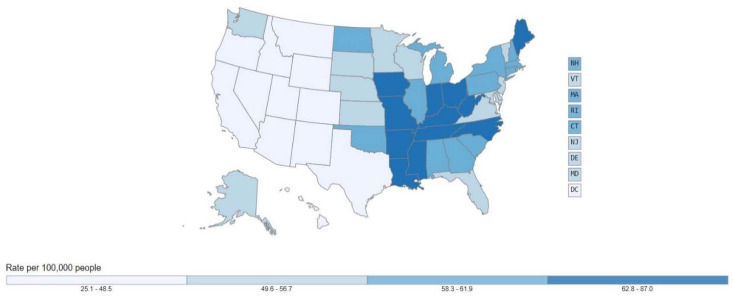
Cancer of the Lung and Bronchus, United States, 2017, Rate per 100,000 people, All ages, all races/ethnicities, Male and Female. Source: Centers for Disease Control and Prevention.

**Figure 2:**
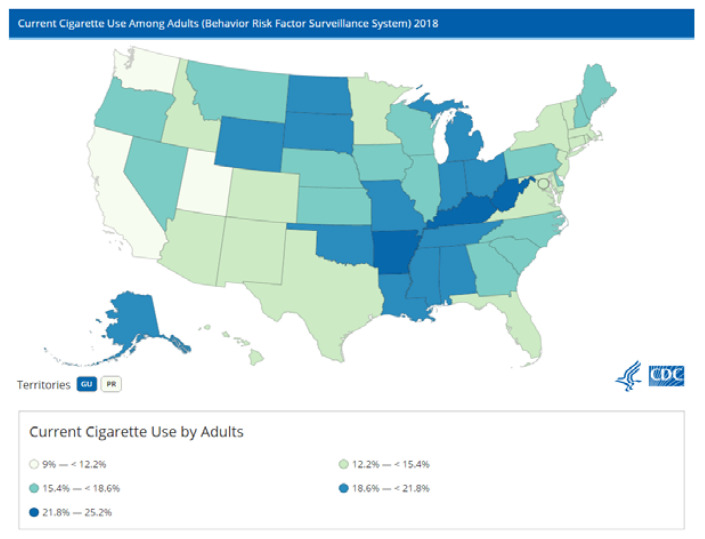
Cigarette Use by Adults, United States, 2018. Source: Centers for Disease Control and Prevention.

There is substantial variation in lung cancer rates within the United States by state. Figure 1 shows lung cancer rates in the United States. Figure 2 shows the adult smoking rates. As can be seen in Figure 1 and Figure 2, the lung cancer rate cannot be attributed solely to adult cigarette usage. Smoking is necessary, but not sufficient, for predicting lung cancer in the United States.

States across the US vary by their presence of polluting industries, which are known to emit ambient emissions hazardous to human health. These ambient emissions include both criteria pollutants, which are more tightly regulated, and hazardous air pollutants, which are less tightly regulated. They also vary in the propensity for state governments to define and enforce environmental protections in order to protect people from breathing ambient emissions. Our research questions are the following:

1. How well does smoking predict lung cancer? How well does state predict it? What other factors should be included?
2. How does a macro-level model (environmental quality) compare to a micro-level model (ambient air pollutants)?
3. What is the best model we can obtain in terms of explanatory power and predictive accuracy?

The purpose of this paper is to investigate and model lung cancer, including cigarette smoking but also other factors, in the United States from 2000 to 2017. By understanding the different contributing factors in different models, we can examine the relative magnitude of their contributions. By doing so, we can discuss the factors amenable to change and how intervention could reduce their impact on lung cancer.

This paper is organized as follows. We first review the literature on lung cancer in the United States, highlighting a variety of factors. We then model lung cancer separately by state, cigarette smoking, environmental quality index, and ambient emissions. We then synthesize the best linear and non-linear model from the simpler models and interpret the results. We then discuss the implications of the model, including possible interventions to decrease the incidence of lung cancer. We conclude with limitations and raise questions for further research.

## 2. Literature Review

Many studies have analyzed the causes of lung cancer, and different approaches have been taken: biological, epidemiological, animal studies, etc. In addition, two types (small cell and non-small cell), which divide into five subtypes of lung cancer have been examined individually or in combination: Small Cell Carcinoma, Combined Small Cell Carcinoma; Adenocarcinoma, Squamous Cell Carcinoma, and Large Cell Carcinoma. Although the different types account for different proportions of lung cancer cases, the consistently largest contributing factor is cigarette smoking. Controlling for smoking, or excluding the smoking factor, has also been researched in multiple ways. We choose in this paper to include cigarette smoking, accounting for it in our models, but also examine other factors in order to compare the magnitudes of influence among the various factors. Ultimately, we combine a variety of factors to arrive at the model that explains the most variance, predicting lung cancer with the greatest accuracy.

Apart from demographic differences, the other contributing factors to lung cancer all pertain to air exposure, either deliberately inhaled (cigarette smoking) or inadvertently inhaled, e.g., diesel exhaust inhaled from cars and trucks. The inadvertent factors include coarse particulate matter, ground-level ozone, sulfur dioxide, and sulfates. In addition to these widely understood factors are ones inhaled without any awareness of doing so: the ambient emissions found in outdoor air and metals or gasses in the ground, e.g., radon in ground soil. We also know that there are interaction effects, in that a smoker exposed to other factors, e.g., asbestos, is particularly prone to developing lung cancer [2]. Some research has developed models of multiple factors as additive, whereas other research studies develop models showing them to be multiplicative, including interaction effects between carcinogens and co-carcinogens [3,4]. 

Table 1 shows some of the mostly influential research studies (average number of citations = 1593), including epidemiological and biological papers, as well as review articles and meta-analyses. The table includes the primary variables examined, the methods used, and the main findings.

For environment quality, we use the environmental quality index (EQI), an umbrella construct which consists of five environmental domains: air, land, water, built environment, and socio-demographic [23,24]. The higher the quintile on each of these domains, the worse the environmental quality. We include all five indices as variables because they account for and aggregate thousands of environmental elements, hundreds of which are potentially carcinogenic. The five domains of the EQI can be useful for spotting broad environmental risks and crafting environmental policies / regulations. The EQI_Air domain variable serves as an approximate aggregation of hundreds of particular metals and gasses, and thus is an overall index, which can be computed, reported, and used as a basis for comparison over time or area: county vs. county or state vs. state. Although the EQI_Air variable is the domain most relevant to this paper, hazardous elements of the environment may also be found within the other four domains.

For the sake of completeness, we include Appendix A, which shows the complete list of 175 metals and gasses tracked by the National Air Toxics Assessment [17]. NATA compiles ambient emissions by geographic unit (county/state) over time, and different counties/states are managed separately, with different regulations and tracking methods. Because of these variations, NATA states that their results “should not be used to quantify benefits of reduced air toxics ambient emissions” [17]. Since we cannot model the complete list of NATA ambient emissions, we develop our own master list of the most hazardous ambient emissions: Cyanide compounds, Carbon Monoxide, Carbon Disulfide, Diesel Exhaust, Nitrogen Dioxide, Tropospheric Ozone, Coarse Particulate Matter, Fine Particulate Matter, and Sulfur Dioxide. One main objective of this paper is to compare models containing these specific ambient emissions (micro variables) versus the macro-level EQI domains (macro variables).

## 3. Materials and Methods

All our data sources are publicly available, consistent with the principle of scientific reproducibility, from which we gathered and checked for data quality. By data quality, we mean correctness (free from errors, noise) and completeness (no missing variables or values). We checked for correctness by checking the plots of the distributions for every variable, looking for impossible or outlying values, which we did not find. Our data sources were already clean, i.e., high quality in that sense. We addressed completeness by (1) integrating data from multiple sources, and (2) imputing for missing values.

Our data sources include multiple providers because triangulation of different sources minimizes biases, assumptions, or blind spots that a particular source may have. Combining disparate sources is likely to yield a unique combination of information, extending and refining our established models to make them more accurate. We used the following four data sources:

- CDC United States Cancer Statistics, 2013–2017
- County Health Ranking Organization, 2011–2013 (University of Wisconsin Population Health Institute)
- EPA Outdoor Air Quality Data, 2006–2010
- Air Quality-Lung Cancer Data, 2000–2005 (National Cancer Institute and Environmental Protection Agency)

Because cancer takes a while to develop in human lungs, from the sustained breathing of harmful ambient emissions to the lung cancer diagnosed years later, we sought data sources spanning a considerable time horizon: 2000–2017. We obtained data at both the county level and state level, aggregating counties to the state level for all fifty states, the unit of analysis. If a state had no value for a county, it was replaced by the state average. Some variables were obtained for two timeframes, in which case we append “_T1” or “_T2” to distinguish them. 

Our data sources for each variable are found in Table 2. The five domain-specific county level environmental quality index (EQI) data values for the period 2000–2005—air, land, water, built environment, and socio-demographic—are abstracted from the United States Environmental Protection Agency profile. Complete descriptions of the datasets used in the EQI are provided in [23].

### 3.1. Data Cleaning

After examining descriptive statistics for each variable, we centered, scaled, and made log transformations for non-normally distribution variables. This was for the purpose of making variables consistent with the assumptions of multiple regression and for decreasing the amount of multi-collinearity. We affix the suffix “_log” to the variable name to indicate a log transformation, e.g., SO2_T1_log and CS2_log. We then checked for outliers and missing values for each variable, and if the proportion of outliers and missing values was less than 10%, replaced them with the median value of each state. If all counties of a state were missing values, those remained NA. The final sample size is 2,862 observations.

Table 3 and Table 4 show the final versions of the variables after cleaning (imputation and/or transformation). Nitrogen Dioxide in 2006–2010 had too many nulls and was therefore excluded from inclusion in any model.

**Figure 3:**
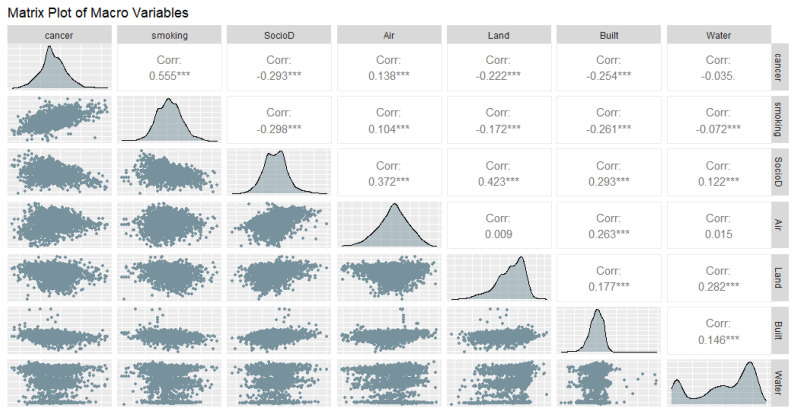
Matrix Plot of Lung Cancer, Adult Smoking, and Environmental Quality Index, all domains. Significance codes: 0 ‘***’ 0.001 ‘.’ 0.1 ‘ ’ 1.

**Figure 4:**
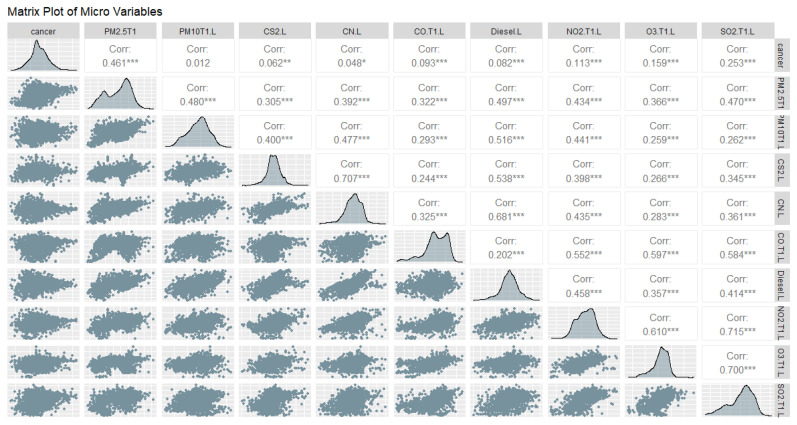
Matrix Plot of Lung Cancer with Variables in Time 1: Particulate Matter 2.5 and 10, Carbon Disulfide, Cyanide compounds, Carbon Monoxide, Diesel Exhaust, Nitrogen Dioxide, Tropospheric Ozone, Sulfur Dioxide. Significance codes: 0 ‘***’ 0.001 ‘**’ 0.01 ‘*’ 0.05 ‘.’ 0.1 ‘ ’ 1.

**Figure 5:**
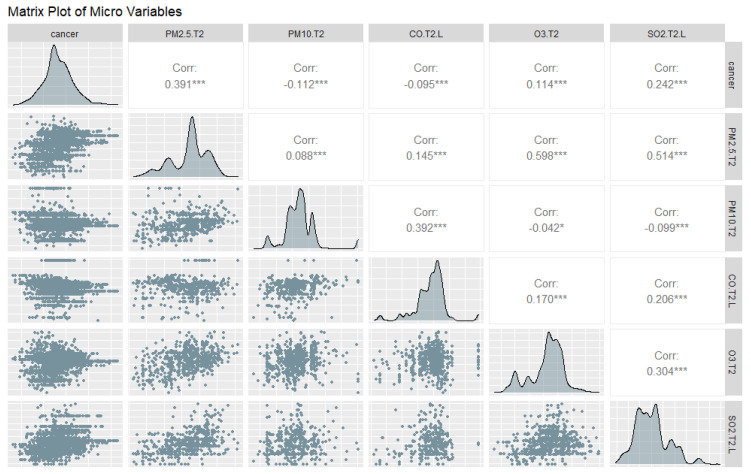
Matrix Plot of Lung Cancer with Micro Variables in Time 2: Particulate Matter 2.5, Particulate Matter 10, Carbon Monoxide, Tropospheric Ozone, Sulfur Dioxide. Significance codes: 0 ‘***’ 0.001 ‘*’ 0.05 ‘.’ 0.1 ‘ ’ 1.

We show a matrix plot among the EQI variables in Figure 3 and a matrix plot among the ambient emissions variables in Figure 4 and Figure 5, to show the correlations at the macro- and micro-levels. Most correlations are significant, which indicates a model is likely to be obtained, but also that we must check for collinearity.

### 3.2. Model Results and Interpretation

Our modelling approach was always the same, regardless of specific method used. We (a) randomly partitioned the dataset into train (80%) and testing (20%) subsets, and (b) checked for outliers, multi-collinearity, and target leakage [25]. Model accuracy was assessed by performance on both a train partition (80%) and test partition (20%), determined by random sampling.

**Figure 6:**
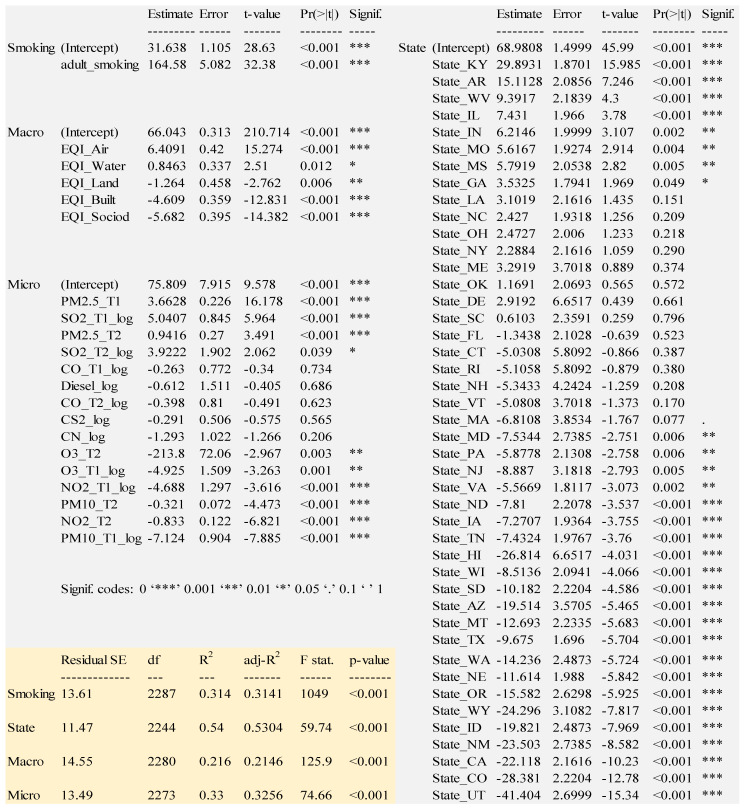
All Regression Models. Significance codes: 0 ‘***’ 0.001 ‘**’ 0.01 ‘*’ 0.05 ‘.’ 0.1 ‘ ’ 1.

We fitted several models starting with two separate layers of variables: (1) adult smoking and (2) states. The rationale for adult smoking is because it is well-established as the number one contributing cause of lung cancer. The rationale for geographic states was because we expected differences by state in terms of ambient emissions, emission regulations, cultural differences, and baseline population health. The geographic states model contains data for forty-five states, using Alabama as the baseline dummy variable state. The remaining five states (Alaska, Kansas, Michigan, Minnesota, and Nevada), five territories (American Samoa, Guam, Northern Mariana Islands, Puerto Rico, Virgin Islands) and Washington D.C. had insufficient data and were therefore excluded from analysis. We then examined models that include (3) only the EQI domain variables and (4) only the ambient emission variables. See Figure 6 for the regression results of the four models.

Smoking is a very strong predictor of lung cancer. For every percentage increase in adult smoking, the number of lung cancer cases increases by 164.583 per 100,000 citizens. The variance explained (adj. R^2^) is 0.3141.

The state model in Figure 6 consists of 45 US states. Figure 6 shows the states sorted by t-value to show the relative magnitude of the impact of state. There are 30 statistically significant states at a level of *p* < 0.05 with all but Georgia significant at a level of *p* < 0.01. Some states have a positive coefficient estimate, indicating a positive association with lung cancer, whereas others have a negative coefficient estimate relative to Alabama, the arbitrary baseline state. The variance explained (adj. R^2^) is 0.5304.

Kentucky has the most positive coefficient, indicating that its citizens have a higher tendency to have lung cancer: 29.893 more cases per 100,000 residents vs. Alabama. There are seven other statistically significant, higher risk states: Arkansas, West Virginia, Illinois, Indiana, Missouri, Mississippi, Georgia. Conversely, Utah has the most negative coefficient, indicating a lower tendency to have lung cancer: 41.404 fewer cases per 100,000 residents vs. Alabama. There are twenty-one other statistically significant (*p* < 0.05), lower risk states: Maryland, Pennsylvania, New Jersey, Virginia, North Dakota, Iowa, Tennessee, Hawaii, Wisconsin, South Dakota, Arizona, Montana, Texas, Washington, Nebraska, Oregon, Wyoming, Idaho, New Mexico, California, and Colorado. Massachusetts is borderline statistically significant (*p* = 0.077).

The macro model consists of only the five EQI variables covering different domains: air, water, land, built, and sociodemographic. We model these by themselves to assess their macro-level impact on lung cancer without any confounding of smoking, state, or ambient emissions. A higher value of each of these indicates worse quality of environment [23]. Figure 6 shows the EQI domains sorted by t-statistic. Positive coefficients indicate worse environmental quality. An EQI_Air coefficient of 6.409 indicates that for every unit of worse air quality, there are 6.409 more lung cancer cases per 100,000 people. Water quality is also positive and statistically significant, but lower impact: 0.846 more lung cancer cases per 100,000 people.

According to the regression coefficients, there are countervailing, counterintuitive forces indicated by the quality of land, socio-demographic, and built domains, because they suggest that areas with *worse* environmental quality in the land, socio-demographic, and built domains have *lower* incidence of lung cancer. Unequal access and socio-economic disparities could partially explain the paradoxical results. Adding higher-order terms was attempted to resolve the paradoxical results, i.e., squared-terms: EQI_Land^2^, EQI_Built^2^, and EQI_SocioD^2^. Interaction terms were also attempted: EQI_Land*EQI_Built, EQI_Land*EQI_SocioD, and EQI_Built*SocioD. None of these higher-order terms helped the interpretability of the coefficients, and they increased the variance explained only a small amount (0.005) while increasing the collinearity, so the higher-order terms were dropped. The variance explained (adj. R^2^) is 0.2146. 

Figure 6 also shows the micro-level variables, nine ambient emissions: Cyanide compounds, Carbon Monoxide, Carbon Disulfide, Diesel Exhaust, Nitrogen Dioxide, Tropospheric Ozone, Coarse Particulate Matter, Fine Particulate Matter, and Sulfur Dioxide. Six of these have data from both timeframes: Carbon Monoxide, Nitrogen Dioxide, Tropospheric Ozone, Coarse Particulate Matter, Fine Particulate Matter, and Sulfur Dioxide. Five of the ambient emissions are statistically significant in both timeframes: Nitrogen Dioxide, Tropospheric Ozone, Course Particulate Matter, Fine Particulate Matter, and Sulfur Dioxide. The higher the level of Fine Particulate Matter or Sulfur Dioxide, the higher the rate of lung cancer. Fine Particulate Matter is the most hazardous in both time periods T1 and T2. Almost as hazardous is Sulfur Dioxide. The variance explained (adj. R^2^) for this model is 0.3256, which is higher than adult smoking by itself.

**Figure 7:**
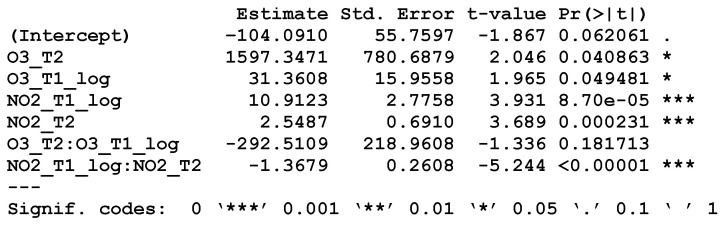
Testing for Interaction.

Paradoxically, the higher the level of Nitrogen Dioxide, Tropospheric Ozone, or Course Particulate Matter, the lower the rate of lung cancer. Lowering the risk, paradoxically, is Course Particulate Matter, which is particular matter up to four times as large as Fine Particulate Matter but still respirable. Coarse Particulate Matter is not healthful, but a larger presence of it could mean that Fine Particulate Matter levels have decreased, amounting to an indirectly positive effect. Similarly, the negative coefficients of Nitrogen Dioxide and Tropospheric Ozone are paradoxical as well, but more difficult to understand. These negative coefficients may indicate countervailing, confounded effects or indirect effects. That is, Nitrogen Dioxide and Tropospheric Ozone may not be the factors directly causing lung cancer. According to Witschi (1988), “there is little evidence to implicate ozone or Nitrogen Dioxide directly as pulmonary carcinogens, but that they might modify and influence the carcinogenic process in the lung.” Overall, Nitrogen Dioxide and Tropospheric Ozone have shown mixed associations with lung cancer, implicated only as co-carcinogens, exacerbating lung disease [26,27,28]. A model testing Tropospheric Ozone and Nitrogen Dioxide in both timeframes with interaction terms results in Figure 7.

The coefficients of Tropospheric Ozone and Nitrogen Dioxide become positive (in both timeframes) in their relationship to lung cancer. The interaction terms are negative, and only the Nitrogen Dioxide interaction term is statistically significant, indicating a dampening multiplicative effect over time. This effect from the Nitrogen Dioxide interaction disappears when the other ambient emissions variables are added back in, so we drop it for the sake of simplicity. We attribute the negative coefficients to complex relationships among the various ambient emissions and possibly other variables not included in our model. These paradoxes notwithstanding, the micro-level model is more comprehensive than the macro-level EQI model. It seems that accounting for exposure to specific carcinogenic ambient emissions is more accurate, capturing more of the variance, than the simpler macro-level model.

**Figure 8:**
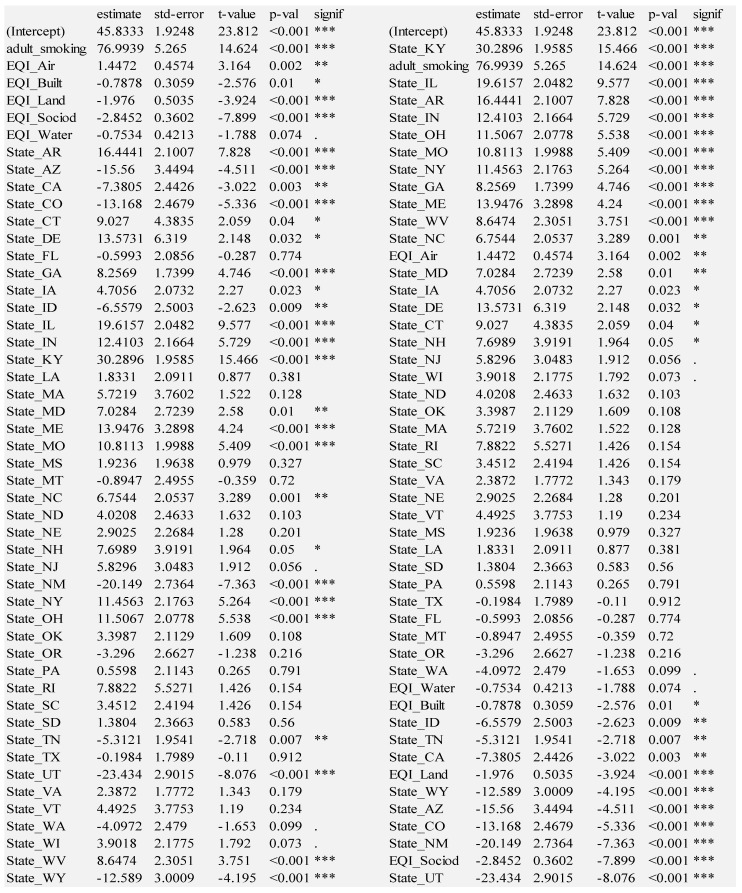
Foundation + Environmental Quality Index; Residual standard error: 10.5 on 2236 degrees of freedom; Multiple R-squared: 0.6197, **Adjusted R-squared: 0.6114**; F-statistic: 74.36 on 49 and 2236 DF, *p*-value: < 2.2 × 10^−16^; Significance codes: 0 ‘***’ 0.001 ‘**’ 0.01 ‘*’ 0.05 ‘.’ 0.1 ‘ ’ 1.

The four models described thus far show significant explanatory and predictive power. We consider the adult smoking and state models to be foundational because adult smoking is obviously crucial to include, and the state model explains the most variance. We therefore combine adult smoking and geographic state to form the foundation for all multi-layer models. We examine the Foundation + EQI model results, grouped by variable layer (left side) and sorted by t-statistic (right side) in Figure 8.

Many states are positively associated with lung cancer, with Kentucky even more hazardous than adult smoking, according to their t-statistics. The next ten states are more hazardous than EQI_Air: Illinois, Arkansas, Indiana, Ohio, Missouri, New York, Georgia, Maine, West Virginia, North Carolina. Note that all of these states are in the Eastern, South, or Midwest regions of the United States. On the other hand, environmental quality indexes of sociodemographic, land, built environment and water domains are negatively associated with lung cancer, which is paradoxical. This could indicate a confounding of unhealthful environmental quality within healthful city living. For example, this could be where lower quality environment (vehicle exhaust) is experienced near high-quality healthcare systems, which can detect lung cancer early. Amidst those environmental domain variables are the states negatively associated with lung cancer: Utah, New Mexico, Colorado, Arizona, Wyoming, California, Tennessee, Idaho. Note that all but Tennessee are states in the Western region of the United States.

### 3.3. Foundation + Ambient Emissions

**Figure 9:**
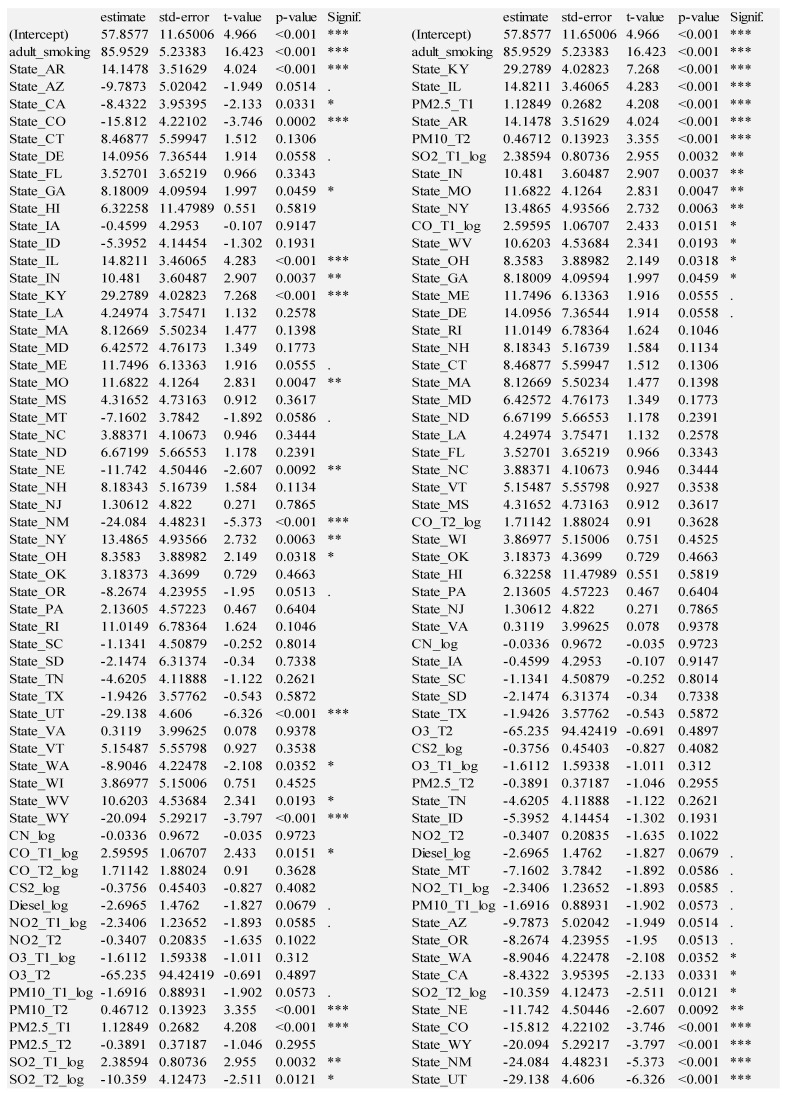
Foundation + Ambient Emissions; Residual standard error: 10.62 on 2228 degrees of freedom; Multiple R-squared: 0.613, **Adjusted R-squared: 0.6026**; F-statistic: 58.82 on 60 and 2228 DF, *p*-value: < 2.2 × 10^−16^; Significance codes: 0 ‘***’ 0.001 ‘**’ 0.01 ‘*’ 0.05 ‘.’ 0.1 ‘ ’ 1.

Next, we show the model combining the foundation with the ambient emissions layer, grouped by variable layer (left side) and sorted by t-statistic (right side) in Figure 9.

In examining the significance of ambient emissions in this model, we see that eight of the fifteen variables are statistically significant. Six of them are from T1, the earlier timeframe: Carbon Monoxide, Diesel Exhaust, Nitrogen Dioxide, Coarse Particulate Matter, Fine Particulate Matter, and Sulfur Dioxide; two are from T2, the later timeframe: Coarse Particulate Matter and Sulfur Dioxide.

Adult smoking regains first place as Kentucky slips to second place. The next two most hazardous states, approximately the same impact as Fine Particulate Matter in T1 are Illinois and Arkansas. Then comes Coarse Particulate Matter in T2 and Sulfur Dioxide in T1 with the following states close behind: Indiana, Missouri, and New York. Then comes CO_T1 and the last three states: West Virginia, Ohio, and Georgia. Note that almost all the hazardous states are in the Midwest or Southern region of the United States. The exception is New York. On the other extreme, Utah still has the lowest rate of lung cancer (29.138 cases fewer per 100,000). The next six least hazardous states are all in the West: New Mexico, Wyoming, Colorado, Nebraska, California, and Washington.

### 3.4. Linear Model of All layers

**Figure 10:**
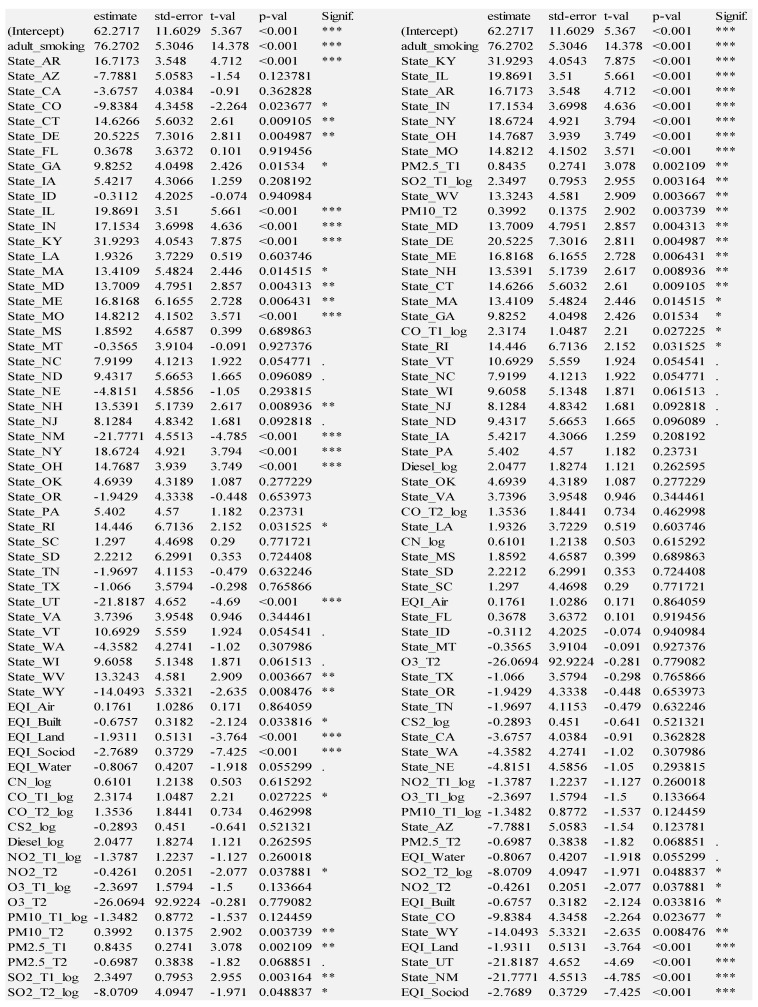
Foundation + EQI + Ambient Emissions; Residual standard error: 10.41 on 2221 degrees of freedom; Multiple R-squared: 0.6285, **Adjusted R-squared: 0.6178**; F-statistic: 58.71 on 64 and 2221 DF, *p*-value: < 2.2 × 10^−16^; Significance codes: 0 ‘***’ 0.001 ‘**’ 0.01 ‘*’ 0.05 ‘.’ 0.1 ‘ ’ 1.

Figure 10 shows the model of all layers, grouped by variable layer (left side), and sorted by t-statistic (right side).

Adult smoking remains the most hazardous variable in the model containing all the layers. The most hazardous states are Kentucky, Illinois, Arkansas, Indiana, New York, Ohio, and Missouri. Then come three ambient emissions: Fine Particulate Matter in T1, Sulfur Dioxide in T1, and Coarse Particulate Matter in T2 with West Virginia in their midst. Finally, the least hazardous states are Maryland, Delaware, Maine, New Hampshire, Connecticut, Massachusetts, Georgia, and Rhode Island. All these states are in the Northeast or Middle Atlantic regions, with the exception of Georgia, which is slightly more hazardous than the effect of Carbon Monoxide in T1. On the other extreme, the biggest impact for reducing the rate of lung cancer is socio-demographic EQI. The least hazardous states are New Mexico, followed by Utah, Wyoming, and Colorado, all in the Western region of the US. Three additional EQI domain variables are healthful: land, built, and water with borderline significance.

The least hazardous variables of smaller impact are Nitrogen Dioxide in T2 and Sulfur Dioxide in T2. They are most likely co-carcinogens, having a negative direct impact, because we know they are hazardous, but indirectly have a beneficial impact on lung cancer. In examining the significance of ambient emissions in the all-layer model, we see that seven of the fifteen are statistically significant. Three of them are from T1, the earlier timeframe: Carbon Monoxide, Fine Particulate Matter, Sulfur Dioxide; four are from T2, the later timeframe: Course Particulate Matter, Sulfur Dioxide, Nitrogen Dioxide and Fine Particulate Matter.

### 3.5. Model Comparison

Whether we choose the macro-, micro-, or combined model, we have a linear model of 61–62% adjusted R-Squared predicting lung cancer. State and adult smoking are the basis for all three models, with state having the largest impact. All the states collectively explain 53.04% of the variance. Adult smoking by itself is the variable with the highest impact, explaining 31.41% of the variance. Adding the macro-level EQI domain variables increases the variance explained to 61.14% of the variance. Adding the micro-level ambient emissions variables instead of the EQI variables increases the variance explained to 60.26%. Including both the macro- and micro-level variables explains 61.78% of the variance. These results suggest that (1) adult smoking is necessary but not sufficient for a good model, and that (2) the macro-, micro-, and combined models have approximately the same power, but achieve it in different ways.

We added several layers of variables and found that the most complete model virtually doubled the variance explained of adult smoking by itself. We also found that macro-variables are a good summary of environmental quality while using only five variables. By using particular ambient emission variables, we achieved the same variance explained, but at the cost of greater complexity. We also found that the state effect closely mirrors the overall rate of lung cancer, regardless of model. States are an interesting, if surprising factor, not one that intuitively comes to mind when predicting lung cancer. State does include many risk factors, however: adult smoking rates (a cultural factor), presence of hazardous industrial ambient emissions (a business factor), government regulation (strong or weak), as well as environmental quality (air and other domains). In terms of ambient emissions, their mix does vary depending on the presence or absence of Environmental Quality Index domains. The strongly significant EQI_Air becomes less significant in the presence of all the particular ambient emission variables. Table 5 summarizes the accuracy metrics for all the linear regression models, both in the train and test partitions (randomly created) of the data.

Model 1 has the best accuracy on five of the seven metrics, whereas model 2 has the best on the remaining two metrics, all indicated in red boldface. In these models, we have seen some paradoxical relationships, i.e., beta coefficients of unexpected sign. Consequently, we tried some more advanced machine learning models to try to improve accuracy and to resolve the paradoxical coefficients. Specifically, we fitted a Ridge Regression, Random Forest, and Gradient Boosted Tree on smoking, state, and EQI variables, with and without ambient emissions. Ridge Regression is worth trying because we have a large number of predictors. Random Forest and Gradient Boosted Trees are methods known to be effective at capturing interactions and/or non-linear relationships between predictors. They do so by aggregating sub-models that have no or low correlation with each other. Because of this, they tend to reduce both errors of bias and errors of variance, which increases overall model accuracy [29,30]. 

The results are found in Table 6. Model 8 has the best accuracy on Root Mean Squared Error (RMSE), the most commonly used metric for prediction, on test data. It is a simple model in that it achieves that accuracy with only smoking, state, and EQI domain variables. Model 9, a Support Vector Machine, does use the emissions variables but is superior only on Mean Absolute Percentage Error (MAPE) of the train partition. Finally, model 10, a Random Forest, uses the emissions variables and is superior on the remaining five metrics, spanning the train and test partitions. We conclude that models 8 and 10 are the best, according to the accuracy metrics in the test partition. We exclude model 9 because it was superior on none of the test data metrics.

If one is required to use a linear model, then models 1 and 2 perform well. They are both dominated by geographic state, however, and some of the variable coefficients are paradoxical. Consequently, we tried to resolve those paradoxes and capture non-linear relationships by fitting advanced machine learning models. Of those models, we arrived at two models, 8 and 10, that perform significantly better than the linear models.

**Figure 11:**
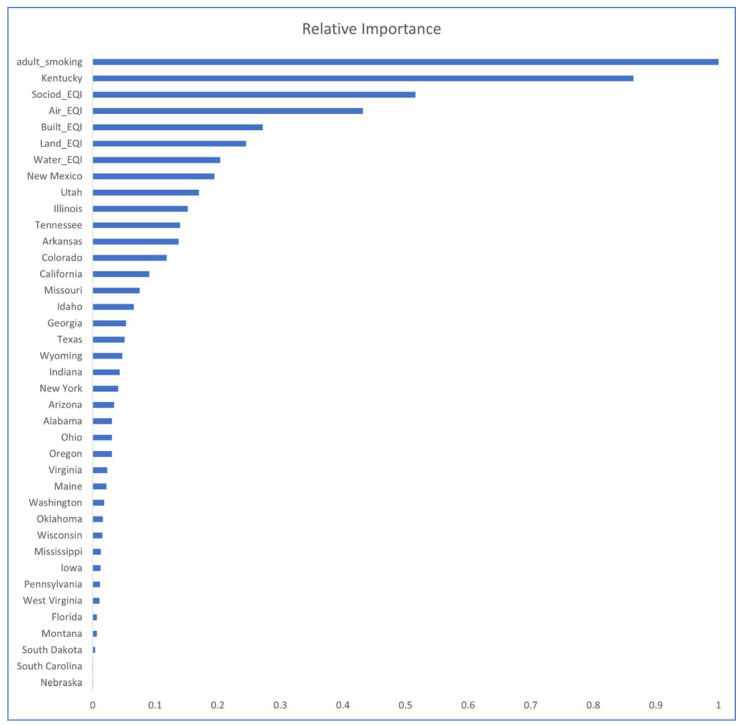
Feature Impact for Model 8: GBT (least squares loss, early stopping), excluding states having no impact.

Figure 11 shows all five EQI domain variables (socio-demographic, air, built, land, and water) among the highest importance predictors, after adult smoking and Kentucky in a Gradient Boosted Tree (model 8). The EQI socio-demographic domain is the only domain with impact higher than that of EQI_ Air. Figure 11 shows adult smoking and Kentucky along with many EQI and ambient emissions: socio-demographic EQI, Fine Particulate Matter (T1 and T2), as well as Carbon Monoxide, Tropospheric Ozone, and Sulfur Dioxide among the highest impact predictors in a Random Forest (model 10). The drawback to these ML models is that they are not as transparent and interpretable as linear models 1 and 2.

**Figure 12:**
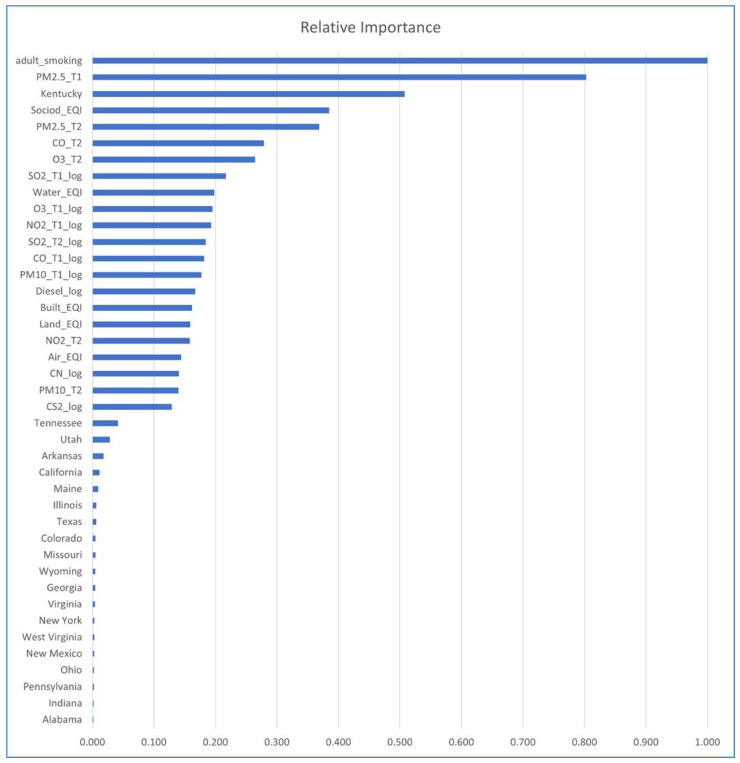
Feature Impact for Model 10: Random Forest (500 trees, terminal node size = 5), excluding states having no impact.

Figure 12 shows the impacts of variables in a Random Forest that includes ambient emissions variables. It concurs with Figure 10 that two of the highest impact variables are adult smoking and Kentucky, but also Particulate Matter 2.5 in T1 and T2. Among the top impact variables are also: EQI sociodemographic and Carbon Monoxide (T2), Tropospheric Ozone (T2), and Sulfur Dioxide (T1). EQI water is the second highest impact EQI domain, whereas EQI_Air drops quite a few places, having been replaced by specific ambient emissions.

We conclude that there is no one conclusively best model to report. Instead, we offer a small set of models to summarize the best, highest performing models (Table 7).

In Table 8, we describe the anthropogenic sources of the highest impact ambient emissions from our best performing models: Fine Particulate Matter, Course Particulate Matter, Sulfur Dioxide, Carbon Monoxide, and Tropospheric Ozone. These hazardous ambient emissions come from a mix of industrial, vehicular, and residential sources. The one common denominator is a burning of fossil fuels.

## 4. Discussion and Contributions

This paper makes several innovative contributions. We combined data from multiple sources in multiple timeframes with multiple methods to predict lung cancer in the United States. We did so in a unique way: by including adult smoking of cigarettes as a base model and then adding several variable layers: state, environmental quality index domains, and ambient emissions. By layering variables and comparing them, we iteratively built strong linear models (variance explained = 61–62%) and strong non-linear models (variance explained = 61-64% with 10% less error). This is the first paper, to the best of our knowledge, to contribute an organized iteration of linear and non-linear models in the lung cancer literature.

State had such a strong impact that we included it with adult smoking of cigarettes as foundational. We found a surprisingly strong variation in the states, with general clustering by region of the United States. States in the Eastern part of the US have significantly higher lung cancer rates than states in the Western United States. We also found that variables reflecting more recent and less recent exposures are both important. Ultimately, we obtain three regression models with variance explained in the range of 61–62%, whether one includes only macro-level variables (EQI), micro-level variables (ambient emissions), or both. Model performance was verified to be strong on multiple metrics in both the train and test dataset partitions.

The EQI variables present a paradox in a simple, linear model. Lower quality air certainly contributes to lung cancer, as does water quality to a lesser degree. The other domains—built environment, sociodemographic, and land—have a negative association with lung cancer. These results could indicate an indirect relationship, in which the direct impact is seemingly healthful, but the larger, indirect impact on lung cancer incidence is a problem. This could also signal, for example, that in older, bigger cities in the East, Midwest, or Southern regions, there is lower quality air from vehicle exhaust or HVAC systems in old buildings, but those cities have other domains that offset the effects of the poor quality air. Note that air and water are the environments that are shared the most, in the public commons. Land and built domains are more privately owned, controlled, and managed.

The model to choose depends on whether one prefers a more interpretable, linear model or a less interpretable, higher performance model that contains linear and non-linear relationships. It also depends on what policies are being examined, macro-level EQI variables or micro-level ambient emissions. We found that the macro-level and micro-level models achieve approximately the same explanatory and predictive power in the linear model. Combining them provides an improvement, particularly in the non-linear models.

The model to choose depends also on one’s specific level of analysis and plans for intervention. For example, if one wanted to introduce broad legislation to improve air quality through taxation, one might prefer the macro-level model. Conversely, if a specific technology designed to control an ambient emission is being deployed, one might prefer the micro-level model. Is one trying to craft/adjust state regulations covering a broad population and range of activities, e.g., industrial ambient emissions, or statewide anti-smoking campaigns? Alternately, is one trying to intervene and strictly limit ambient emissions, such as the ones we found most hazardous across the models: Fine Particulate Matter, Coarse Particulate Matter, Sulfur Dioxide, and Carbon Monoxide? Finally, is one trying to limit ambient emissions know to be co-carcinogenic, e.g., Tropospheric Ozone and Nitrogen Dioxide, because they can facilitate and accelerate the damage of carcinogens past the possibility of early detection and treatment?

Methodologically, we encounter a tradeoff question. How much transparency are we willing to give up in exchange for greater accuracy in our models? This is the ongoing dilemma of Machine Learning and Artificial Intelligence. Our machine learning models improve by 1–2% on the variance explained (R^2^) and they shrink the error metrics (RMSE, MAE, and MAPE) by approximately 10 percent. These tradeoffs need to be assessed by policy makers according to their use cases and impacts on various stakeholders. Policymakers need to, at the very least, show that these models commit no ethical violation, i.e., no discrimination against protected classes of people (race, ethnicity, gender, etc.). Ideally, we would be able to open the best ML/AI “black-box,” through Explainable Artificial Intelligence (XAI) methods to understand and communicate how all linear and non-linear relationships have been captured.

## 5. Limitations and Directions for Future Research

We need to investigate the paradox in which harmful ambient emissions have a negative regression coefficient rather than a positive one. At face value, this would indicate a hazardous inhalable emission that is good for human health. This is clearly impossible, and it represents a paradox in need of untangling. We also acknowledge that gender and race also play a role in predicting the prevalence of lung cancer. Some occupations are predominantly held by men, e.g., coal mining, where air quality is a known hazard. Future research could incorporate data on occupational hazards, gender, and race to extend and refine our model.

Health insurance coverage, its cost, and proximity to high quality healthcare vary geographically. Some states have more stringent smoke-free air laws designating some areas, e.g., workplaces, as smoke-free. Other states have more vigorous smoking cessation programs to help smokers quit. Healthcare to treat lung cancer also varies in quality and effectiveness by geography. Some states also have a greater proportion of industries that emit hazardous ambient emissions or air quality regulation enforcement that may be lax. In this study, we account for these various factors only by geographic state since that is our level and unit of analysis. Further research could refine our models by examining the US by county. Doing so could examine geographic proximity to high quality care and health insurance coverage/cost by demographic variables, which vary widely within states. Alternatively, we could subdivide into 374 Metropolitan Statistical Area (MSA) or 955 Core-Based Statistical Area (CBSA). Analyzing at the county, MSA, or CBSA level would be more granular.

According to U.S. Census data and Simmons National Consumer Survey (NHCS), in 2020, 510,000 Americans smoked two or more packs per day. The cost of cigarettes varies considerably—a pack costs $6–$10—indicating a substantial financial burden that varies by socio-economic class. In 2020, it was reported that the average cost for a pack of cigarettes across the US was $6.28, but higher state and local taxes increase that to $10.67 in New York City. A single pack-a-day habit in New York amounted to $3895 per year. This of course may discourage the initiation of cigarette smoking or encourage smokers to finally quit. Many adults have quit smoking, a difficult achievement, thus improving their health and reducing second-hand smoke for those around them. They have also saved their families substantial amounts of money and reduced the burden on the healthcare system. The prevalence of cigarette smoking varies by gender, race, state, region, and socio-economic class. These differences ought to be studied in further research, perhaps stratified into several categories of smoker: everyday smoker, someday smoker, former smoker, never smoker.

Finally, future research could use classification methods to predict high vs. low lung cancer rates. This would require determination of the proper cutoff between high and low classes. Then we could compare logistic regression vs. classification trees, random forests, and other methods. Accuracy would be determined by sensitivity, specificity, F1 statistic, and AUC/ROC. These models could focus on the predictor variables found to have the highest relative impact in models 8 and 10: adult smoking, state (or other geographic unit), EQI domains, Particulate Matter 2.5, Carbon Monoxide, Tropospheric Ozone, and Sulfur Dioxide.

## 6. Conclusions

Cigarette smoking is known to contribute to lung cancer. The individual choice whether to smoke is thus a key predictor of lung cancer, as our models show. Less well-known is that some geographic states are positively associated with lung cancer, e.g., Kentucky, and other states are negatively associated, e.g., Utah. States are an interesting bundle of factors that contribute to lung cancer because they encapsulate choices made by individuals, businesses, industries, and government leaders. States also differ in their environmental quality in several domains: air, water, built, land, and socio-demographic.

Results from our best models show that all five EQI domains are highly significant. Results from our best models show that these are the most significant ambient emissions: Particulate Matter 2.5, Carbon Monoxide, Tropospheric Ozone, and Sulfur Dioxide. These were found to be important over multiple timeframes. State policies, regulations, and restrictions could make a difference in the mitigation of these ambient emissions to reduce the rate of lung cancer. The linear models have approximately 62% of the variance explained and highlight many states that contribute to or protect against lung cancer. In addition, the models show the harmful influence of Particulate Matter 2.5, Sulfur Dioxide, Carbon Monoxide, and Particulate Matter 10, as well as the protective influence of socio-demographic, land, and built domains of the environment. The best machine learning model (a Random Forest) captures 64% of the variance explained, with approximately 10% less error.

In our best linear and non-linear models, we see the importance of all five Environmental Quality Index domains. We also see the impact of several ambient emissions. The common denominator for addressing all the hazards is the need to reduce burning of fossil fuels. As we transition from fossil fuels to renewable fuels, we will need to revisit these models. Future research could also improve our models by including data regarding occupational hazards, demographics, and socio-economics, as well as by subdividing state into county or other more granular units.

## Figures and Tables

**Table 1:** Literature Review.

ID	Variables	Methods/Data Source(s)	Findings
[5]	Fine particulates, including sulfates	Regression, 14–16 year mortality follow-up of 8111 adults in 6 US cities/Prospective cohort study	After adjusting for smoking, mortality strongly associated with air pollution with fine particulates
[6]	Race and socioeconomic and gender predictors of early-state non-small cell lung cancer	Regressions/SEER	Higher socioeconomic status helps survival, as does being Caucasian or female.
[7]	PM_10_, SO_4_, SO_2_, O_3_, and NO_2_ checked for lung cancer	6,338 nonsmoking, non-Hispanic white SDA residents of California were enrolled in 1977/Adventist Health Study (AHS)	Levels of PM_10_, SO_4_, SO_2_, O_3_, NO_2_ far higher for those with lung cancer, especially in males.
[8]	PM_2.5_ and SO_2_, lung cancer, lung cancer mortality	Cox Proportional hazards model/American cancer society, part of cancer prevention study (CPS-II), ongoing prospective mortality study of 1.2 M adults	PM_2.5_ and SO_2_ associated with lung cancer; each 10 microgram/m3 increase associated with 8% increase in lung cancer mortality
[9]	Race, gender, SE class, chemicals, not just smoking	Datasets from SEER and NPCR/National Cancer Institute’s Surveillance, Epidemiology, and End Results (SEER) Program and the Centers for Disease Control and Prevention’s National Program of Cancer Registries (NPCR)	Epidemiologically and biological studies show strong causation between smoking and cellular mutations; racial disparities: Black worst, then white, then other races; lower Socio-Economic class is strongly associated with lung cancer; not gender; race seems to be proxy for Socio-Economic class
[10]	carbon dioxide, ozone, cancer, Ozone Mortality, Ozone Hospitalization, Ozone Emergency Room Visits, and Particulate Matter Mortality pollution mortality	Mathematical model/Nasa and EPA and California air resources	A climate-air pollution model showed by cause-and-effect analysis that fossil-fuel CO2 increases U.S. surface ozone, carcinogens, and Coarse Particulate matter, increasing cancer rates
[11]	asbestos fibers and ambient Coarse Particulate Matter PM_10_, PM_2.5_ and diesel exhaust particles	Chemicals purchased and combined with smoke, passed through filters/experiments	Synergistic effects in the generation of hydroxyl radicals in smoke with environmental asbestos fibers and ambient PM_10_, PM_2.5_ and diesel exhaust particles (DEP). The highest synergistic effects were observed with the asbestos fibers, PM_2.5_ and DEP, producing redox recycling and oxidative action.
[12]	Ozone and PM_2.5_ to predict premature (excess) mortality	Simulations of preindustrial and present-day (2000) concentrations included rural areas/epidemiology literature	Tropospheric O_3_ and PM_2.5_ contribute substantially to global premature mortality from lung cancer, which is 14% higher than baseline.
[13]	Socioeconomic, Rural-Urban, and Racial Inequalities in US Cancer Mortality:	Stats (regression)/three national data sources: the national mortality database, the decennial census, and the 2009–2010 Area Resource File	Blacks experiencing higher mortality from each cancer than whites within each deprivation group. Socioeconomic gradients in mortality were steeper in non-metropolitan than in metropolitan areas. Mortality disparities may reflect inequalities in smoking and other cancer-risk factors, screening, and treatment.
[14]	All of them	Statistics	Intersectionality of all the variables
[15]	PM_2.5_ and O_3_	80,285 AHSMOG-2 participants were followed for an average of 7.5 years; Logistic regression/Adventist Health and Smog Study-2 (AHSMOG-2), a cohort of health-conscious nonsmokers, where 81% have never smoked.	Lung cancer is associated with PM_2.5_ in never smokers and slightly higher if 1+ hrs./Day outdoors or 5+ years at residence.
[16]	Cancer risk index (CRI) Incidence of cancer risk from air toxics	Statistical modelling of San Antonia Texas; racial disparities found/Data for CRI from National Air Toxics Assessment [17]	Cancer risk index is all positively correlated with the ambient diesel coarse particulate matter. Institutional transformations are essential to mitigate the social-ecological divide.
[18]	Radon, Lung cancer	Meta-analysis of 8 case-control studies of indoor radon, where *n* = 200+/Finland (2), USA (2), Sweden (2), China, Canada	Relative risk is 14% greater for those exposed to indoor radon versus the controls
[19]	Occupational lung cancer, asbestos, arsenic, chromium, radon, silica, beryllium, nickel, cadmium, diesel exhaust	Review of many studies of workers in the U.S.	Conservative estimates are that relative risk of occupational lung cancer is 1.31 for diesel fumes, 2.0 for asbestos, and 3.69 for arsenic; several million exposed workers in early 1980 s
[20]	24 experts in a working group	Review of many studies: human, occupational, outdoor, indoor, animal.	From many sources, respirable PM_10_, PM_2.5_, NO_2_, SO_2_, and O_3_ are frequently and substantially above safe levels. Consistency in studies shows cellular damage, as well as genetic and epigenetic effects.
[21]	Demographics, cancer types, cigarette features all lead to mutations and other changes in the genes	Review of smoking: all epidemiologically and biological studies show strong causation, and it parallels the rise and fall of cigarette smoking/Many sources	Prevention important and cessation important because it causes cancer in all demographics. Stopping smoking is the most important cause of lung cancer.
[22]	Incidence and survival of Small-Cell Lung Cancer among all lung cancers by Gender and Smoking and Stage of cancer	Analysis of the Surveillance, Epidemiologic, and End Results (SEER) database	Proportion of SCLC has diminished, and survival has increased slightly, attributed to decreasing smoking and increased proportion of low-tar cigarettes

**Table 2:** Variables and their Descriptions, Timeframe, Data source.

Var.	Description	Time	Data Source
New Case of Lung Cancer	Cancer of the Lung or Bronchus, All Ages, All Races/Ethnicities, Male and Female. Rate per 100,000 people	2013–2017 (mean)	CDC United States Cancer Statistics
Adult Smoking	Percentage of adults who are current smokers (county level)	2011–2013 (mean)	County Health Ranking Organization
Land EQI	Environmental Quality Index–Land Domain	2000–2005 (mean)	Air Quality-Lung Cancer Data
SocioD EQI	Environmental Quality Index–Socio-Demographic Domain	2000–2005 (mean)	Air Quality-Lung Cancer Data
Built EQI	Environmental Quality Index–Built Environment Domain	2000–2005 (mean)	Air Quality-Lung Cancer Data
Air EQI	Environmental Quality Index–Air Domain	2000–2005 (mean)	Air Quality-Lung Cancer Data
Water EQI	Environmental Quality Index–Water Domain	2000–2005 (mean)	Air Quality-Lung Cancer Data
PM2.5_T1	Fine Particulate Matter (2.5 micrometers or smaller) Mean of 24 h period	2000–2005 (mean)	Air Quality-Lung Cancer Data
PM10_T1	Coarse Particulate Matter (10 micrometers or smaller) based on Mean of 24 h period	2000–2005 (mean)	Air Quality-Lung Cancer Data
SO2_T1	Sulfur Dioxide	2000–2005 (mean)	Air Quality-Lung Cancer Data
NO2_T1	Nitrogen Dioxide	2000–2005 (mean)	Air Quality-Lung Cancer Data
CO_T1	Carbon Monoxide	2000–2005 (mean)	Air Quality-Lung Cancer Data
O3_T1	Tropospheric (ground level) Ozone	2000–2005 (mean)	Air Quality-Lung Cancer Data
CN_T1	Cyanide compounds	2000–2005 (mean)	Air Quality-Lung Cancer Data
Diesel	Gaseous exhaust produced by a diesel type of internal combustion engine	2000–2005 (mean)	Air Quality-Lung Cancer Data
CS2	Carbon Disulfide	2000–2005 (mean)	Air Quality-Lung Cancer Data
PM2.5_T2	Fine Particulate Matter (2.5 micrometers or smaller), weighted annual mean (mean weighted by calendar quarter), based on weighted mean 24 h	2006–2010 (mean)	EPA Outdoor Air Quality Data
PM10_T2	Coarse Particulate Matter (10 micrometers or smaller), weighted annual mean (mean weighted by calendar quarter), based on weighted mean 24 h	2006–2010 (mean)	EPA Outdoor Air Quality Data
SO2_T2	Sulfur Dioxide Mean 1 h (the annual mean of all the 1-h measurements in the year)	2006–2010 (mean)	EPA Outdoor Air Quality Data
NO2_T2	Nitrogen Dioxide Mean 1 h (the annual mean of all the 1-h measurements in the year)	2006–2010 (mean)	EPA Outdoor Air Quality Data
CO_T2	Carbon Monoxide 2nd Max 8 h (the 2nd highest non-overlapping 8-h avg in the year)	2006–2010 (mean)	EPA Outdoor Air Quality Data
O3_T2	Tropospheric Ozone 4th Max 8 h, the 4th highest daily max 8-h average in the year	2006–2010 (mean)	EPA Outdoor Air Quality Data

**Table 3:** Variables and Data Cleaning.

Variable	Description	Imputation	Transformation
New Cases of Lung Cancer	Cancer of the Lung/Bronchus, Rate per 100,000 people	none	none
Adult Smoking	Percentage of adults who are current smokers	none	none
PM2.5_T1	Particulate Matter 2.5 in Time 1	none	none
PM10_T1_log	Particulate Matter 10 in Time 1	none	Logarithm
SO2_T1_log	Sulfur Dioxide in Time 1	none	Logarithm
NO2_T1_log	Nitrogen Dioxide in Time 1	none	Logarithm
CO_T1_log	Carbon Monoxide in Time 1	median	Logarithm
EQI_Land	Environmental Quality Index, Land Domain	median	none
EQI_SocioD	Environmental Quality Index, Socio-Demographic Domain	none	none
EQI_Built	Environmental Quality Index, Built Domain	none	none
O3_T1_log	Tropospheric Ozone in Time 1	none	Logarithm
CN_log	Cyanide compounds	none	Logarithm
Diesel_log	Diesel Exhaust	none	Logarithm
CS2_log	Carbon Disulfide	none	Logarithm
EQI_Air	Environmental Quality Index, Air Domain	none	none
EQI_Water	Environmental Quality Index, Water Domain	none	none
PM2.5_T2	Particulate Matter 2.5 in Time 2	none	none
PM10_T2	Particulate Matter 10 in Time 2	median	none
SO2_T2_log	Sulfur Dioxide in Time 2	none	Logarithm
CO_T2	Carbon Monoxide in Time 2	none	none
O3_T2	Tropospheric Ozone in Time 2	median	none
NO2_T2	Nitrogen Dioxide in Time 2	---------	---------

**Table 4:** Descriptive Statistics.

Var. Type	Variable	Description	Min.	1 Q	Median	Mean	3 Q	Max.
Target	Lung Cancer	Lung/Bronchus Cancer Rate	14.600	56.800	65.360	66.220	75.700	132.400
Baseline	Adult Smoking	Current Adult Smokers (%)	0.000	0.173	0.207	0.210	0.243	0.425
Macro Variables	EQI_Air	Environmental Quality Index, Air Domain	−2.532	−0.349	0.177	0.147	0.692	2.508
	EQI_Built	Environmental Quality Index, Built Domain	−3.993	−0.408	0.177	0.119	0.672	7.283
	EQI_Land	Environmental Quality Index, Land Domain	−3.149	−0.395	0.207	0.078	0.672	2.095
	EQI_SocioD	Environmental Quality Index, Socio-Demographic Domain	−3.331	−0.584	0.022	0.027	0.570	3.979
	EQI_Water	Environmental Quality Index, Water Domain	−1.701	−0.614	0.359	0.063	0.889	1.478
Micro Variables	CN_log	Cyanide compounds	−3.743	−2.118	−1.812	−1.842	−1.523	−0.022
	CO_T1_log	Carbon Monoxide	0.650	2.248	2.555	2.503	2.944	3.800
	CO_T2	Carbon Monoxide	0.267	1.191	1.558	1.691	1.900	7.020
	CS2_log	Carbon Disulfide	−6.900	−3.875	−3.436	−3.429	−2.975	0.361
	Diesel_log	Diesel Exhaust	−1.773	−0.711	−0.526	−0.539	−0.356	0.495
	NO2_T1_log	Nitrogen Dioxide	1.306	2.383	2.657	2.632	2.905	3.818
	NO2_T2	Nitrogen Dioxide	1.000	7.811	8.700	9.231	11.125	24.400
	O3_T1_log	Tropospheric Ozone	2.341	3.456	3.641	3.598	3.810	4.876
	O3_T2	Tropospheric Ozone	0.053	0.069	0.072	0.071	0.075	0.090
	PM10_T1_log	Particulate Matter 10	1.030	2.129	2.452	2.406	2.692	3.678
	PM10_T2	Particulate Matter 10	10.000	19.420	21.990	22.210	23.700	40.200
	PM2.5_T1	Particulate Matter 2.5	2.167	7.940	10.417	9.941	11.782	16.912
	PM2.5_T2	Particulate Matter 2.5	4.500	9.743	11.171	10.855	12.419	17.150
	SO2_T1_log	Sulfur Dioxide	0.251	1.679	2.154	2.035	2.478	3.569
	SO2_T2_log	Sulfur Dioxide	1.000	22.000	33.000	36.980	49.000	98.000

**Table 5:** LR: Linear Regression; RMSE: Root Mean Squared Error; MAE: Mean Absolute Error; MAPE: Mean Absolute Percentage Error.

ID	Meth.	Variable Groups	TRAIN (80%)				TEST (20%)		
			adj. R^2^	RMSE	MAE	MAPE	RMSE	MAE	MAPE
1	LR	smoking + state + EQI + emissions	0.617	10.067	7.507	12.133	11.155	8.281	13.901
2	LR	smoking + state + EQI	0.612	10.168	7.556	12.241	11.167	8.259	13.858
3	LR	smoking + state + emissions	0.602	10.273	7.697	12.478	11.416	8.579	14.332
4	LR	state	0.527	11.239	8.198	13.324	11.792	8.664	14.414
5	LR	emissions	0.322	13.543	10.494	17.089	13.996	10.818	18.316
6	LR	smoking	0.308	13.724	10.367	17.401	14.777	11.083	19.289
7	LR	EQI	0.211	14.639	11.098	18.633	15.297	11.429	20.338

**Table 6:** RF: Random Forest, GBT: Gradient Boosted Tree; RR = Ridge Regression; SVM = Support Vector Machine; RMSE: Root Mean Squared Error; MAE: Mean Absolute Error; MAPE: Mean Absolute Percentage Error.

ID	Meth.	Variable Groups	TRAIN (80%)				TEST (20%)		
			adj. R^2^	RMSE	MAE	MAPE	RMSE	MAE	MAPE
8	GBT	smoking + state + EQI	0.611	10.340	7.611	12.334	9.976	7.377	12.054
9	SVM	smoking + state + EQI + emissions	0.634	10.026	7.335	11.833	10.027	7.401	12.063
10	RF	smoking + state + EQI + emissions	0.639	9.960	7.268	11.926	10.068	7.314	11.977
11	GBT	smoking + state + EQI + emissions	0.625	10.151	7.445	12.132	10.239	7.535	12.252
12	RR	smoking + state + EQI + emissions	0.600	10.486	7.741	12.667	10.314	7.822	12.881
13	RR	smoking + state + EQI	0.598	10.507	7.758	12.688	10.322	7.793	12.784
14	RF	smoking + state + EQI	0.584	10.684	7.814	12.932	10.383	7.627	12.570

**Table 7:** Best performing Models: Variance Explained, Root Mean Squared Error.

Predictor Variables	Linear Model (adj. R^2^, RMSE)	Non-Linear Model (adj. R^2^, RMSE)
smoking + state + EQI	Linear Regression (0.612, 11.167)	Gradient Boosted Tree: (0.611, 9.976)
smoking + state + EQI + Emissions	Linear Regression (0.617, 11.155)	Random Forest (0.639, 10.068)

**Table 8:** Anthropogenic Sources of the Highest Impact Ambient Emissions.

Ambient Emission	Anthropogenic Sources
Particulate Matter	Combustion of carbon-based fuels. Smokestacks; power plants, automobiles. Diesel- and gasoline-powered motor vehicles and equipment; burning wood in residential fireplaces, wood stoves, wildfires, agricultural and other fires. Cement dust, fly ash, oil smoke, and smog from construction sites, unpaved roads and fields [31].
Sulfur Dioxide	Fuel combustion in mobile sources, e.g., automobiles, locomotives, ships, and other equipment; burning of fossil fuels (coal, oil, and diesel) or other materials that contain sulfur at power plants and other industrial facilities. Smelting of mineral ores (aluminum, copper, zinc, lead, and iron) that contain sulfur. Eastern states have more sulfate particles than the West, mostly because of sulfur dioxide emitted by large, coal-fired power plants [32].
Carbon Monoxide And Tropospheric Ozone	Burning of fossil fuels (gasoline, natural gas, oil, coal, and wood) in vehicles or machinery. Poorly vented gas appliances (furnaces, ranges, ovens, water heaters, clothes dryers, etc.), many in the home: Fireplaces, wood, and gas stoves Coal or oil furnaces Space heaters or oil or kerosene heaters Charcoal grills, camp stoves Gas-powered lawn mowers and power tools Automobile exhaust fumes Portable generator Leaking chimneys Cigarettes, pipes, and cigars smoked in the home. Carbon monoxide can also react with other gases to form Tropospheric Ozone. Carbon monoxide detectors should be installed in everyone’s home near any garage, combustion equipment, and bedroom.

## Data Availability

The data that support the findings of this study are available from the corresponding author, upon reasonable request.

## Appendix A. Emissions tracked by the National Air Toxics Assessment

1,1,1-Trichloroethane	Acetaldehyde	Chlorobenzilate	Hexachlorobutadiene	O-Toluidine
1,1,2,2-Tetrachloroethane	Acetamide	Chloroform	Hexachlorocyclopentadiene	Pah/Pom
1,1,2-Trichloroethane	Acetonitrile	Chloromethyl Methyl Ether	Hexachloroethane	Parathion
1,1-Dimethylhydrazine	Acetophenone	Chloroprene	Hexamethylene Diisocyanate	P-Dioxane
1,2,3,4,5,6-Hexachlorocyclohexane	Acrolein	Cobalt	Hexamethylphosphoramide	Pentachloronitrobenzene
1,2,4-Trichlorobenzene	Acrylamide	Coke Oven Emissions	Hexane	Pentachlorophenol
1,2-Dibromo-3-Chloropropane	Acrylic Acid	Cresols	Hexavalent Chromium	Phenol
1,2-Diphenylhydrazine	Acrylonitrile	Cumene	Hydrazine	Phosgene
1,2-Epoxybutane	Allyl Chloride	Cyanide	Hydrochloric Acid	Phosphine
1,2-Propylenimine	Aniline	Dibenzofuran	Hydrogen Fluoride	Phosphorus
1,3-Butadiene	Antimony	Dibutyl Phthalate	Hydroquinone	Phthalic Anhydride
1,3-Dichloropropene	Arsenic	Dichloroethyl Ether	Iodomethane	Polychlorinated Biphenyl
1,3-Propane Sultone	Benzene	Dichlorvos	Isophorone	P-Phenylenediamine
1,4-Dichlorobenzene	Benzidine	Diesel Pm10	Lead	Propionaldehyde
2,2,4-Trimethylpentane	Benzotrichloride	Diethanolamine	Maleic Anhydride	Propoxur
2,4,5-Trichlorophenol	Benzyl Chloride	Diethyl Sulfate	Manganese	Propylene Dichloride
2,4,6-Trichlorophenol	Beryllium	Dimethyl Phthalate	Mercury	Propylene Oxide
2,4-Dichlorophenoxyacetic Acid	Biphenyl	Dimethyl Sulfate	Methanol	Quinoline
2,4-Dinitrophenol	Bis(2-Ethylhexyl) Phthalate	Dimethylcarbamoyl Chloride	Methoxychlor	Quinone
2,4-Dinitrotoluene	Bis(Chloromethyl) Ether	Epichlorohydrin	Methyl Chloride	Selenium
2,4-Toluenediisocyanate	Bromoform	Ethyl Acrylate	Methyl Isobutyl Ketone	Styrene
2-Acetylaminofluorene	Bromomethane	Ethyl Carbamate	Methyl Isocyanate	Styrene Oxide
2-Chloroacetophenone	Cadmium	Ethyl Chloride	Methyl Methacrylate	Tetrachloroethylene
2-Nitropropane	Calcium Cyanamide	Ethylbenzene	Methyl Tert-Butyl Ether	Titanium Tetrachloride
3,3’-Dichlorobenzidine	Captan	Ethylene Dibromide	Methylene Chloride	Toluene
3,3’-Dimethoxybenzidine	Carbaryl	Ethylene Dichloride	Methylhydrazine	Toluene-2,4-Diamine
3,3’-Dimethylbenzidine	Carbon Disulfide	Ethylene Glycol	N,N-Dimethylaniline	Toxaphene
4,4’-Diphenylmethane Diisocyanate	Carbon Tetrachloride	Ethylene Oxide	N,N-Dimethylformamide	Trichloroethylene
4,4’-Methylenebis(2-Chloroaniline)	Carbonyl Sulfide	Ethylene Thiourea	Naphthalene	Triethylamine
4,4’-Methylenedianiline	Catechol	Ethylenimine	Nickel	Trifluralin
4,6-Dinitro-O-Cresol	Chloramben	Ethylidene Chloride	Nitrobenzene	Vinyl Acetate
4-Aminobiphenyl	Chlordane	Formaldehyde	N-Nitrosodimethylamine	Vinyl Bromide
4-Dimethylaminoazobenzene	Chlorine	Glycol Ethers	N-Nitrosomorpholine	Vinyl Chloride
4-Nitrobiphenyl	Chloroacetic Acid	Heptachlor	N-Nitroso-N-Methylurea	Vinylidene Chloride
4-Nitrophenol	Chlorobenzene	Hexachlorobenzene	O-Anisidine	Xylene
